# Clinical and economic burden of surgical complications during hospitalization for digestive cancer surgery in *France*

**DOI:** 10.1002/cnr2.1895

**Published:** 2023-10-02

**Authors:** Guillaume Piessen, Catherine Dive‐Pouletty, Aurélie Danel, Magali Laborey, Benoît Thomé

**Affiliations:** ^1^ Service de Chirurgie Digestive et Oncologique CHU Lille Lille France; ^2^ CNRS, Inserm, CHU Lille, UMR9020‐U1277‐CANTHER–Cancer, Heterogeneity Plasticity and Resistance to Therapies University of Lille Lille France; ^3^ Nestlé Health Science Issy‐les Moulineaux France; ^4^ Nestlé Health Science Vevey Switzerland; ^5^ Median Conseil Pau France

**Keywords:** burden, digestive cancer surgery, infection

## Abstract

**Background:**

Surgical complications and particularly infections after digestive cancer surgery remain a major health and economic problem and its burden in France is not well documented.

**Aims:**

The aim of this study was to analyse recent data regarding surgical complications in patients undergoing major digestive cancer surgery, and to estimate its burden for the French society.

**Methods and Results:**

Using the 2018 French hospital discharge database and 2017 National CostStudy we studied hospital stays for surgical resection in patients withdigestive cancer. The population was divided into three groups based onpostoperative outcomes: no complications (NC), related infectious complications (RIC) and other complications. The main analysis compared the length and cost per stay between RIC and NC. Forty‐Four thousand one hundred and twenty‐three stays following a digestive cancer resection were identified. Lower gastro‐intestinal cancers were the most prevalent representing 74.8% of stays, the rate of malnutrition was 32.8% and 15.8% of patients presented RIC. Mean (SD) length of stay varied from 11,7 (9.0) days for NC to 25,5 days (19.5) for RIC (p < 0.01). The mean cost per patients' stay (SD) varied from €10 641 (€ 5897) for the NC to €18 720 (€7905) for RIC (*p* < .01).

**Conclusion:**

The risk of RIC after digestive cancer resection remains high (>15%) and was associated with significantly longer length of stay and higher cost per stay. Although important prevention plans have been implemented in recent years, care strategies are still needed to alleviate the burden on patients and the healthcare system.

## 1 INTRODUCTION

In 2016, the estimated number of healthcare‐associated infections (HAIs) in Europe was 8.9 million, including 4.5 million in acute care hospitals.^1^ The infection prevalence in France was estimated at 7.6% in 2017 versus 5.6% in 2012 after surgery with no specific data for digestive surgery.^2^ Reducing HAIs at hospital has been a public health priority for many years as they can result in a significant increase of the length of hospital stay,^3^, ^4^, ^5^, ^6^ a complicated drug treatment, repeated surgical interventions, an increased risk of mortality^7^ and issues related to antimicrobial resistance.^8^ The 2017 annual epidemiological report of the European Centre for Disease Prevention and Control indicated that the surgical site infection (SSI) percentage was influenced by the type of procedure, with the highest rate of 10.1% SSIs reported for colon surgeries.^9^ Consistently, a study conducted in UK hospitals from 2013 to 2018 reported that colon surgery presented the highest cumulative incidence of SSIs with a percentage of 8.7%.^10^ A French study carried out in 2006 in patients (*N* = 330) hospitalized for digestive surgery highlighted a prevalence of 5.9% of SSIs for digestive surgery, and 15.9% for digestive cancer surgery,^11^ while a study conducted in Japan showed a prevalence of 10.7% of post‐operative infections in patients who underwent digestive surgery.^12^ Another French study analyzing patients identified with ICD10 codes of cancer and digestive surgery act‐procedures from 2012 to 2016 from the national health database named “Echantillon généraliste des bénéficiaires” reported that 46% of patients have infectious complications.^13^

Therefore, recommendations to reduce post‐operative infections were proposed.^14^, ^15^, ^16^, ^17^, ^18^, ^19^ In France, a national nosocomial infection control program was established in 1995 and updated over the years.^20^, ^21^ Despite all groups, programs, and recommendations, the prevalence of infections remains high. Recent data from the UK and France even suggest increased rates in recent years up to 60% after large and small bowel and digestive surgeries.^2^, ^22^ Studies are available on the total economic burden^23^ however, none has assessed the incidence of infections and its economic consequences after surgery according to the location of digestive cancers.

As many protocols have been put in place to improve recovery and reduce complications after digestive cancer surgery in France, the aim of this study was to provide updated figures on their burden.

## 2 MATERIALS AND METHODS

### 2.1 Data source

This study was a retrospective analysis using the 2018 French National hospital discharge database for medicine, surgery, and obstetrics (Programme de Médicalisation des Systèmes d'Information—Médecine, Chirurgie et Obstétrique, PMSI‐MCO). This medico‐administrative database gathers medico‐administrative information for all patients admitted to public and private hospitals in France until their discharge. The primary aim of this database is to finance hospitals with a classification system as American hospitals do with their diagnosis‐related group (DRG); it summaries discharge information like number of patients, number and duration of stays per patient. The International Classification of Diseases, 10th revision (ICD‐10) is used for the medical information. Medical procedures using the common classification of medical acts (CCAM) which is a classification of all procedures performed by medical doctors in hospitals, are coded for all stays. The severity of stays is also coded using a medico‐economic model which considers the healthcare resource utilization for each DRG. Severity ranges from no severity (0) to high severity (4).

A quality control of hospital coding is audited annually by health authorities for a sample of hospitals.

### 2.2 Study population

Forty‐Four thousand one hundred and twenty‐three stays have been analyzed. Mean (SD) age was 69.2 (12.4) years old and the men proportion was 57% (Table 1). The study was performed on an extraction of this database which is all the hospital stays of patients who were hospitalized in 2018 for surgery for digestive cancer using ICD‐10 codes for cancers (from C15 to C26 as Principal or Related Diagnosis) and CCAM codes for digestive surgery procedures: esophagectomy, gastrectomy, segmental resection of duodenum, pancreatectomy, hepatectomy, splenectomy, and biliary tract surgery; and segmental resection of the small bowel, colectomy and proctectomy. No exclusion criteria were applied. Subgroups were defined depending on the digestive location of the cancer: upper gastro‐intestinal (GI), hepato‐pancreato‐biliary, and lower GI. The CCAM codes and subgroups used for the inclusion criteria and for the subgroups were reviewed by a digestive surgeon (GP).

**TABLE 1:** Characteristics of the study population.

	NC	%^a^	RIC	%^a^	*p* value^b^	C‐RIC	%^a^	OC	%^a^	*p* value^b^	Total	%^a^
*N* [%]^c^	33 164 [75.2]	100	6971 [15.8]	100	/	4948 [11.2]	100	3988 [9.0]	100	/	44 123 [100]	100
Age, mean (SD)	69.2 (12.4)	/	69.2 (12.4)	/	/	69.3 (12.3)	/	69.6 (12.2)	/	/	69.2 (12.4)	
Men, *n*	18 364	55.4	4314	61.9	/	3051	61.7	2467	61.2	/	25 145	57,0
Women, *n*	14 800	44.6	2657	38.1	/	1897	38.3	1521	38.8	/	18 978	43.0
Malnutrition *n*	9305	28.1	3494	50.1	<.01	2507	50.1	1692	42.4	<.01	14 491	32.8
Severity level^b^, *n*												
1	8976	27.1	9	<0.1	<.01	7	<0.1	41	1.0	<.01	9026	20.5
2	9806	29.6	138	2.0		11	0.2	1282	32.1		11 226	25.4
3	10 820	32.6	3137	45.0		2349	47.5	1562	39.2		15 519	35.2
4	3516	10.6	3683	52.8		2578	52.1	1099	27.6		8298	18.8
J	30	0.1	–		/	–		1	0.0	/	31	<0.1
Z	16	0.0	4	0.1	/	3	0.1	3	0.1	/	23	<0.1
Death, *n*	704	2.1	399	5.7	<.01	296	6.0	251	6.3	<.01	1354	3.1
Transferred to ICU, *n*	18 127	54.7	5271	75.6	<.01	3779	76.4	2838	71.2	<.01	26 236	59.5

Abbreviations: C‐RIC, confirmed‐related infectious complication; ICU, intensive care unit; NC, no complication; OC, other complication; RIC, related infectious complication.

^a^ %: The proportion within the *N* of the first line (33 164: NC; 6072: RIC; 4948 confirmed RIC; 3988 OC; 44 123 total stays).

^b^ *p* versus NC, chi‐squared contingency table tests.

^c^ [%]: The proportion of the population within the line.

Then, three groups of hospital stays were defined according to the complication after surgery: stays with related infectious complication (RIC), stays with other complication (OC) meaning a non‐infectious complication, and stays without complication (NC) using diagnosis of additional complication classification to compare them. The RIC group was defined by the ICD‐10 code T81.4 “Infection following a procedure, not elsewhere classified”, or by a diagnosis of additional complication with ICD‐10 related to infectious pathologies (see Additional file 1: List of 48 pathologies) combined with a complication code (T81.8, T81.9, T81.7). The OC group was defined by stays with codes related to a complication and already selected in the RIC groups (details of coding in the Appendix). Finally, the NC group included patients not part of the two previous groups. A sub‐group of RIC has been defined: “The confirmed‐related infectious complication group (C‐RIC)” was a subgroup of RIC including only the ICD‐10 code T81.4.

Coding at hospital was done under the supervision of a medical doctor expert in coding. As we were interested in infections directly related to surgery, this study did not include re‐admission for infection. Of note, PMSI database do not collect infections occurring at home after hospital discharge.

### 2.3 Costs calculation

For each DRG, a cost is associated by the French agency in charge of hospital funding (ATIH). Costs of the patients groups of interest were calculated using the 2017 National Cost study method (Echelle Nationale de Coûts à Méthodologie Commune, ENCC), and not using the tariff method, as per the French National Authority for Health (Haute Autorité de Santé) recommendation.^24^ In the ENCC, the cost is established using a representative sample of about 135 hospitals (public and private) which represents 17% and 8% of stays in public and private hospitals in France, respectively, yet with correct to good statistical estimates according to ATIH as the hospital sample represent a majority of hospital stays. In the ENCC model all the costs per stay are included, e.g. staff resource used, equipment, drug, and support services. All these costs are consolidated into Diagnosis related groups (DRG) costs.

### 2.4 Statistical analysis

Descriptive analysis was provided with mean, standard deviation for all quantitative variables (age, LOS, cost). Percentages were calculated for qualitative variables (sex, severity, malnutrition, cancer location, transfer to ICU). Average cost per stay and length of stay comparison between groups were performed using unpaired Wilcoxon test because the assumptions for using the t‐test were not met as the standard deviations of costs and length of stay by patient group or by patient group and digestive site were not similar. Analyses were performed with R Studio software 3.6.1. The dispersion of costs showing quartiles was represented using boxplot.

## 3 RESULTS

**FIGURE 1:**
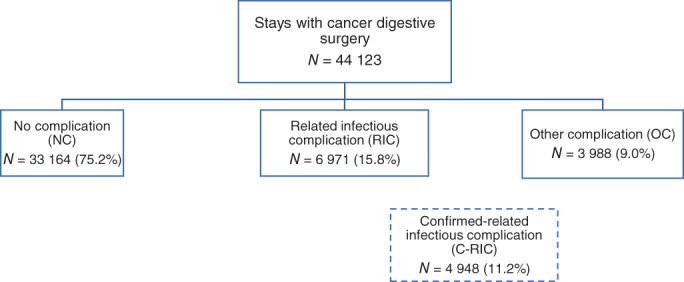
Number of stays per patient group.

Overall, 44 123 stays for digestive cancer surgery were included (Figure 1). The rate of stays was: RIC: 15.8% (C‐RIC: 11.2%), OC: 9%, and NC: 75.2% (Table 1). Surgery of tumor located on the lower GI tract was the most frequent and concerned 74.8% of the stays (Table 2) Malnutrition was present in 32.8% of stays and was more frequent in RIC and OC groups when compared with the NC group (50.1% and 42.4% vs. 28.1% [*p* < .01 and *p* < .01] respectively). The RIC group presented a higher severity: 52.8% of stays with RIC were associated with the highest severity level (number 4) versus 10.6% in the NC group (*p* < .01). 75.6% of patients with RIC were transferred to intensive care during the stay compared with 54.7% in the NC group (*p* < .01), and the death rate was at least twice higher in groups with complications (RIC and OC) compared with NC (*p* < .01).

**TABLE 2:** Description of location of malignant neoplasm in study population.

NC			RIC			C‐RIC	OC			Total	
*N* [%]^a^	33 164 [75.2]	%^b^	6971 [15.8]	%^b^	*p* value^c^	4948 [11.2]	3988 [9.0]	%^b^	*p* value^c^	44 123	%^b^
Upper GI	3649 [69.6]	11.0	1113 [21.2])	16.0	<.01	755 [14.4]	479 [9.1]	12.0	.034	5241	11.9
Esophagus	468 [55.4]	1.4	284 [33.6]	4.1	<.01	192 [22.7]	93 [11.0]	2.3	<.01	845	1.9
Stomach	2257 [71.6]	6.8	622 [19.7]	8.9	<.01	417 [13.2]	275 [8.7]	6.9	.409	3154	7.1
Small intestine	867 [73.9]	2.6	202 [17.2]	2.9	.086	143 [12.2]	104 [8.9]	2.6	.476	1173	2.7
Other	57 [82.6]	0.2	5 [7.2]	0.1		3 [4.3]	7 [10.1]	0.2		69	0.2
Hepato pancreato biliary	3946 [67.1]	11.9	1129 [19.2]	16.2	<.01	746 [12.7]	802 [3.6]	20.1	<.01	5877	13.3
Pancreas	1825 [64.3]	5.5	607 [21.4]	8.7	<.01	397 [14.0]	407 [14.3]	10.2	<.01	2839	6.4
Gallbladder	323 [85.4]	1.0	30 [7.9]	0.4		20 [5.3]	25 [6.6]	0.6		378	0.9
Liver and intrahepatic bile ducts	1492 [71.7]	4.5	302 [14.5]	4.3	.218	201 [9.7]	286 [13.8]	7.2	<.01	2080	4.7
Other and unspecified parts of biliary tract	306 [52.8]	0.9	190 [32.8]	2.7	<.01	128 [22.1]	84 [14.5]	2.1	<.01	580	1,3
Lower GI	25 569 [77.5)	77.1	4729 [14.3]	67.8	<.01	3447 [10.4]	2707 [8.2]	67.9	<.01	33 005	74.8
Colon	18 054 [79.2]	54.4	2966 [13.1]	42.5	<.01	2149 [9.4])	1764 [7.7]	44.2	<.01	22 784	51.6
Rectosigmoid junction	2019 [78.1]	6.1	342 [13.2]	4.9	<.01	245 [9.5]	223 [8.6]	5.6	.095	2584	5.9
rectum	5363 [72.5]	16.2	1357 [18.3]	19.5	<.01	1002 [13.5]	678 [9.2])	17.0	.107	7398	16.8
Anus and canal anal	133 [55.6]	0.4	64 [26.8]	0.9		51 [21.3]	42 [17.6]	1.1		239	0.5

Abbreviations: C‐RIC, confirmed‐related infectious complication; GI, Gastrointestinal tract; NC, no complication; OC, other complication; RIC, related infectious complication.

^a^ [%] represents the proportion of the population within the line.

^b^ %: The proportion within the N of the first line (33 164: NC; 6072: RIC; 4948 confirmed RIC; 3988 OC; 44 123 total stays).

^c^ *p* versus NC, Fisher's exact test.

The mean length of stay and the mean cost per stay was significantly greater than NC, regardless of type of complication (*p* < .01, Tables 3 and 4). The length of stay was increased by a factor of 2.2 (11.7 vs. 25.5 days, *p* < .01) and the hospital stay cost by 75.9% (€ 10 641 vs. € 18 720, *p* < .01) when comparing the RIC group to the NC group (Table 3).

**TABLE 3:** Hospital cost and length of stay by patients group.

Total stays (public and private)	Number of stays	Length of stay, mean (SD)	*p* value test^a^	Total costs (€)	Cost per stay (€), mean (SD)	*p* value test^a^
No complication (NC)	33 164	11.7 (9.0)	–	352 085 564	10 641 (5897)	–
Related infectious complication (RIC)	6971	25.5 (19.5)	<0.01	130 178 411	18 720 (7905)	<.01
Of which confirmed‐related Infectious complication (C‐RIC)	4948	26.7 (19.9)		91 398 748	18 528 (7736)	
Other complication (OC)	3988	17.8 (14.2)	<0.01	58 281 235	14 636 (7592)	<.01
Total	44 123	14.4 (12.9)		540 545 210	12 278 (7092)	

Abbreviation: SD, standard deviation.

^a^ *p* versus NC.

**TABLE 4:** Hospital cost and length of stay by patients group and digestive site.

Total stays (public and private)	Number of stays	Length of stay, mean (SD)	*p* value test^a^	Total costs (€)	Cost per stay (€), mean (SD)	*p* value test^a^
**Upper GI**	**5241**	**18.2 (16.0)**		**85 633 878**	**16 355 (9260)**	
No complication (NC)	3649	14.1 (10.9)		50 182 824	13 764 (7919)	
Related infectious complication (RIC)	1113	30.2 (22.0)	<0.01	26 565 794	23 912 (9016)	<.01
Of which confirmed‐related infectious complication (C‐RIC)	755	32.0 (23.1)		17 794 098	23 631 (8980)	
Other complication (OC)	479	21.3 (16.9)	<0.01	8 885 260	18 550 (9114)	<.01
Hepato pancreato biliary	5877	17.4 (15.4)		94 451 962	16 274 (8453)	
No complication (NC)	3946	13.7 (10.2)		54 129 900	13 912 (7227)	
Related infectious complication (RIC)	1129	29.0 (23.0)	<0.01	25 855 153	23 168 (8277)	<.01
Of which confirmed‐related infectious complication (C‐RIC)	746	30.2 (23.2)		16 702 661	22 725 (8218)	
Other complication (OC)	802	19.5 (14.9)	0.012	14 466 908	18 152 (8490)	<.01
**Lower GI**	**33 005**	**13.3 (11.6)**	**–**	**360 459 370**	**10 928 (5803)**	**–**
No complication (NC)	25 569	11.0 (8.4)		247 772 840	9697 (4922)	
Related infectious complication (RIC)	4729	23.6 (17.6)	<0.01	77 757 465	16 450 (6362)	<.01
Of which confirmed‐related infectious complication (C‐RIC)	3447	24.8 (17.9)		56 901 988	16 517 (6339)	
Other complication (OC)	2707	16.7 (13.3)	0.136	34 929 066	12 908 (6288)	<.01
Total	44 123	14.4 (12.9)		540 545 210	12 278 (7092)	

Abbreviation: GI, gastrointestinal tract; SD, standard deviation.

^a^ *p* versus NC.

**FIGURE 2:**
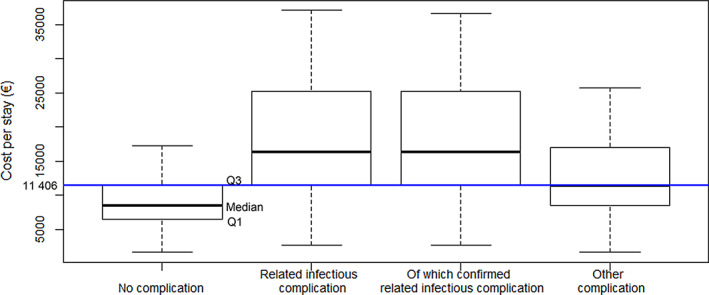
Boxplot of cost per hospital stay by patient group.

With regards to the dispersion of cost per stay, 75% of the stays of the NC group cost lower than 75% of the RIC and C‐RIC groups, as the third quartile (Q3) of NC stay cost (€ 11 406) was similar to the first quartile (Q1) of RIC and C‐RIC groups (Figure 2). Furthermore, 50% (median) of stays with OCs costed more than €11 406.

The significant increase in the length of stay and the cost per stay in RIC (confirmed in C‐RIC) and OC groups versus NC was observed regardless of the digestive location, except for length of stay in lower GI when comparing the OC group versus the NC group (p = 0.136). In every digestive site, there was at least a two‐fold increase in length of stay and in cost between the NC and RIC groups (confirmed in C‐RIC) (Table 4).

## 4 DISCUSSION

This study provides updated numbers of incidence of complications, including infections of the surgery for digestive cancer and new numbers on their associated economic burden. The analysis was performed on the PMSI database which gathered all French hospitalizations occurring during the year 2018 providing a full picture of the rate of infection post digestive surgery which has been coded.

In this study the incidence appears to be stable compare with a previous study suggesting a plateau effect in reducing these complications: 15.8% of RIC observed in this study is similar to 15.9% observed by Minchella et al., in 2008.^11^ The difference observed with the study carried out by Challine et al., in 2019 (46%)^13^ can be explained by the fact that only infectious complications related to digestive oncological surgery were considered in our study, while all infectious complications were considered in the study by Challine et al.

Similarly, our results on the duration of hospitalization are of the same order of magnitude as those previously published. In the literature, an increase of length of stay related to SSI has been reported, varying from 10 to 16.8 days according to the type of surgery that was considered.^5^, ^13^, ^25^ Accordingly in the current study, the mean additional length of stay was 13.8 days for RIC (15 days for C‐RIC). Furthermore, the increase in mean hospital cost per stay was € 8079 for RIC (and € 7887 for C‐RIC), in line with previous studies reporting extra cost from € 6100 to € 18 300.^5^, ^25^

This study highlights the high incremental cost of € 56 318 709, (mean cost for NC 18720—mean cost for RIC 10641)* number of RIC stays i.e. 6971) and confirms that this burden ranging from €8079 (costs of RIC vs. NC) to €3995 (cost of OC vs. NC) is high regardless of the location (Table 3).

A French study published in 2013 by Lamarsalle et al.^26^ reported a threefold increase of length of stay due to HAIs after surgery, while a two‐fold increase in this study was observed. However, the length of stay observed with HAIs was similar: 22 and 25.5 days. Lamarsalle et al. also estimated the additional annual cost to be € 57 892 715 related to infection which was similar to the additional cost observed for RIC in our study (€ 56 318 709).

The advantage of a retrospective analysis based on PMSI database is that it summarizes the information related to healthcare resource used for all public and private hospital stays, hence eliminating the sampling errors. No linkage with external datasets is required to provide the data, hence eliminating linkage difficulties. Coding errors are expected to be rare, as this database constitutes the prospective financial system based on hospital activity and financial resources are directly related to the coded information. However, as a common rule, the quality of the coding increases when the code related to a major feature of the stay (e.g. surgical procedures, digestive cancer in this study) or to a severity characteristic (e.g. infections) because it increases the funding of the stay, which is the case for most of the codes used to define RIC and C‐RIC groups. Furthermore, to prevent miscoding and potential underestimation of SSI at hospital, stays with infection but not confirmed as “related infection due to surgery” were included in the stud.

This study focused on SSIs only reported during the hospital stay with digestive cancer surgery. Consequently, the two SSIs reported after hospital discharge and readmission were excluded which is a limitation of this study. According to a systematic review investigating the proportion of SSI occurring after hospital discharge, 9.9% of surgeries leading to SSI and 60.1% of SSI appeared after discharge.^27^ A U.S. study^28^ concluded that patients with post‐operative infections where about five times more likely to be readmitted to hospital than those with no complication. Outpatients diagnosed with SSI have more frequent readmissions than unaffected patients (27.8% vs 6.8% respectively), and these readmissions are on average 2 days longer in duration than readmissions for other causes.^29^, ^30^ Hence, proportions of SSI and associated additional cost have certainly been underestimated in this study.

Several international working groups published recommendations to reduce surgical complication rates, enhance functional recovery, and reduce hospital length of stay and cost, especially for digestive surgery.^14^, ^15^, ^16^, ^18^, ^19^ The ERAS® Society (Enhanced Recovery After Surgery) was created in 2001 to develop perioperative care and to improve recovery through research, education, audit, and implementation of evidence‐based practice. National guidelines were also published by French scientific societies in digestive surgery in 2005 and in 2014.^14^, ^15^, ^16^, ^18^, ^19^

Monitoring systems and control program^20^, ^21^ have contributed to a decrease of the prevalence of healthcare‐associated infections in France between 1996 and 2012. Based on the first French Prevalence Survey Study conducted in 1996^31^ 6.7% of hospitalized patients had at least one nosocomial infection against 5.1% in the 2012 study.^32^ The proportion of SSI among HAI has increased from 10.6% in 1996 to 13.5% in 2012^31^, ^32^ and now, it seems that a threshold has been reached. Despite the existing international recommendations and French guidelines, nosocomial infections and particularly SSIs remain a major health problem in France. The prevalence of infected patients has remained stable since 2012, with 4.98% of patients with at least one HAI per year; nevertheless, the proportion of SSIs has increased by 2.4 point in 5 years to reach 15.9% of HAIs in 2017.^2^ In view of this progression and given the high clinical and economic burden of infectious complications in patients with digestive surgery, training programs for healthcare professionals and implementation of international recommendations in the current practice should be continued to reduce the SSI rates and their detrimental consequences. Given the significant differences between the non‐complication and the complications patients groups, it is relevant to contemplate enhanced perioperative recovery programs and immunonutrition when they apply. Enhanced perioperative recovery programs and immunonutrition could alleviate clinical and economic burdens of SSIs associated with digestive cancer surgery and its consequences.^15^, ^16^, ^19^ Impact of prehabilitations programs have been proposed but with no current validate evidence at present.^33^ Additionally, institution of integrated practice units, which are practices that organize care delivery through multidisciplinary teams consisting of surgeons, nurses, case managers, technicians, dieticians, psychologists, and others can have a large impact on patients outcomes. Such teams can include infection control specialists to monitor patient status during and after the surgery.^34^

## 5 CONCLUSIONS

This study confirmed that the risk of related infectious complications after digestive cancer resection remains high in France (>15%) and showed that those complications are associated with significantly longer length of stay and higher cost per stay. Although important prevention plans have been implemented in the past years, there is still a need for care strategies to alleviate the burden on the patients and healthcare system.

## AUTHOR CONTRIBUTIONS

**Guillaume Piessen:** Writing – review and editing (equal). **Catherine Dive‐Pouletty:** Supervision (equal). **Aurélié Danel:** Supervision (supporting). **Magali Laborey:** Writing – original draft (equal). **Benoît Thomé:** Formal analysis (supporting); methodology (lead); supervision (lead); writing – review and editing (equal).

## CONFLICT OF INTEREST STATEMENT

Piessen receiving consulting fees from Astellas, Bristol Myers Squibb, Medtronic, and Nestlé Health Science; Benoît Thomé is the founder and CEO of Median Conseil, has worked with several clients tied to the healthcare or nutrition domains, namely Grunenthal, Lundbeck, Octapharma, Kiowa, Baxter, Physidia, Nestlé, Alexion, Amgen, Ipsen, Mallinkrodt, Merck, Pharma Mar, Takeda, Thea, 3M, and Urgo.

## ETHICS STATEMENT

No ethical approval was needed from IRB for this study as it was carried out within a special procedure defined by the French personal data protecting authority (CNIL) called “méthodologie de référence 006”. This procedure allows the automatic right to carry out a study provided the stakeholders fulfill given requirements.

## Data Availability

The data that support the findings cannot be shared as it is forbidden by French registration of PMSI.

## APPENDIX A Selection of codes. Clinical and economic burden of surgical complications during hospitalization for digestive cancer surgery in France

### A.1 Stays for digestive cancer

Main diagnosis or related diagnosis (diagnostic principal, diagnostic relié)

C15*, C16*, C17*, C18*, C19*, C20*, C21*, C22*, C23*, C24*, C25*, C26*

+

One procedure among the following

**Table:**

HEFA020	OEsophagectomie totale sans rétablissement de la continuité, par cervicotomie et par laparotomie
HEFA004	OEsophagectomie totale avec oesophagogastroplastie, par cervicotomie et par laparotomie
HEFA006	OEsophagectomie totale avec oesophagocoloplastie, par cervicotomie et par laparotomie
HEFA008	OEsophago‐pharyngo‐laryngectomie totale avec oesophagogastroplastie, par cervicotomie et par laparotomie
HEFA017	OEsophago‐pharyngo‐laryngectomie totale avec oesophagocoloplastie, par cervicotomie et par laparotomie
HEFA022	OEsophagectomie totale sans rétablissement de la continuité, par thoracotomie
HEFA001	OEsophagectomie avec oesophagogastroplastie, par thoracophrénotomie gauche
HEFA013	OEsophagectomie avec oesophagogastroplastie, par thoraco‐phréno‐laparotomie
HEFA003	OEsophagectomie avec oesophagogastroplastie, par thoracotomie et par coelioscopie
HEFA012	OEsophagectomie avec oesophagogastroplastie, par thoracotomie et par laparotomie
HEFA018	OEsophagectomie avec oesophagogastroplastie, par cervicotomie, thoracotomie et coelioscopie
HEFA002	OEsophagectomie avec oesophagogastroplastie, par cervicotomie, thoracotomie et laparotomie
HEFA016	OEsophagectomie avec oesophagocoloplastie, par thoraco‐phréno‐laparotomie
HEFA009	OEsophagectomie avec oesophagocoloplastie, par thoracotomie et par laparotomie
HEFA007	OEsophagectomie avec oesophagocoloplastie, par cervicotomie, thoracotomie et laparotomie
HEFA005	OEsophagectomie avec oesophagojéjunostomie, par thoraco‐phréno‐laparotomie
HEFA011	OEsophagectomie avec oesophagojéjunostomie, par thoracotomie et par laparotomie
HFFC001	Résection partielle atypique de la paroi de l'estomac n'interrompant pas la continuité, par coelioscopie
HFFA009	Résection partielle atypique de la paroi de l'estomac n'interrompant pas la continuité, par laparotomie
HFFA003	Gastrectomie partielle supérieure [polaire supérieure] avec rétablissement de la continuité, par laparotomie
HFFC012	Gastrectomie partielle inférieure avec anastomose gastroduodénale, par coelioscopie
HFFA002	Gastrectomie partielle inférieure avec anastomose gastroduodénale, par laparotomie
HFFC002	Gastrectomie partielle inférieure avec anastomose gastrojéjunale, par coelioscopie
HFFA006	Gastrectomie partielle inférieure avec anastomose gastrojéjunale, par laparotomie
HFFC017	Gastrectomie totale avec rétablissement de la continuité, par coelioscopie
HFFA005	Gastrectomie totale avec rétablissement de la continuité, par laparotomie
HFFA008	Dégastrogastrectomie partielle avec rétablissement de la continuité, par laparotomie
HFMA005	Totalisation secondaire de gastrectomie avec rétablissement de la continuité, par laparotomie
HGPA001	Duodénotomie à visée thérapeutique ou duodénectomie partielle, par laparotomie
HGCA007	Exclusion du duodénum, par laparotomie
HGMA002	Remise en circuit secondaire du duodénum, par laparotomie
HNFC028	Pancréatectomie gauche avec conservation de la rate, par coelioscopie
HNFA008	Pancréatectomie gauche avec conservation de la rate, par laparotomie
HNFA002	Pancréatectomie gauche avec conservation de la rate, avec anastomose pancréatojéjunale ou pancréaticojéjunale, par laparotomie
HNFC002	Pancréatectomie gauche avec splénectomie [Splénopancréatectomie gauche], par coelioscopie
HNFA013	Pancréatectomie gauche avec splénectomie [Splénopancréatectomie gauche], par laparotomie
HNFA010	Pancréatectomie gauche avec splénectomie [Splénopancréatectomie gauche] avec anastomose pancréatojéjunale ou pancréaticojéjunale, par laparotomie
HNFA001	Isthmectomie pancréatique avec rétablissement de continuité du conduit pancréatique, par laparotomie
HNFA011	Pancréatectomie totale ou subtotale avec conservation du duodénum, sans splénectomie, par laparotomie
HNFA006	Pancréatectomie totale ou subtotale avec conservation du duodénum et splénectomie, par laparotomie
HNFA007	Duodénopancréatectomie céphalique, par laparotomie
HNFA004	Duodénopancréatectomie totale avec splénectomie [Splénopancréatectomie totale], par laparotomie
HGFA001	Résection de l'angle duodénojéjunal avec rétablissement de la continuité, par laparotomie
HGFA005	Résection segmentaire unique de l'intestin grêle pour occlusion, par laparotomie
HGFC014	Résection segmentaire unique de l'intestin grêle sans rétablissement de la continuité, en dehors de l'occlusion, par coelioscopie
HGFA003	Résection segmentaire unique de l'intestin grêle sans rétablissement de la continuité, en dehors de l'occlusion, par laparotomie
HGFC021	Résection segmentaire unique de l'intestin grêle avec rétablissement de la continuité, en dehors de l'occlusion, par coelioscopie
HGFA007	Résection segmentaire unique de l'intestin grêle avec rétablissement de la continuité, en dehors de l'occlusion, par laparotomie
HGFC016	Résection segmentaire multiple de l'intestin grêle, par coelioscopie
HGFA004	Résection segmentaire multiple de l'intestin grêle, par laparotomie
HGFA013	Résection totale de l'intestin grêle, par laparotomie
HHFA026	Colectomie droite sans rétablissement de la continuité, par laparotomie
HHFA009	Colectomie droite avec rétablissement de la continuité, par laparotomie
HHFA008	Colectomie droite avec rétablissement de la continuité, par coelioscopie ou par laparotomie avec préparation par coelioscopie
HHFA018	Colectomie transverse, par laparotomie
HHFA023	Colectomie transverse, par coelioscopie ou par laparotomie avec préparation par coelioscopie
HHFA014	Colectomie gauche sans libération de l'angle colique gauche, sans rétablissement de la continuité, par laparotomie
HHFA017	Colectomie gauche sans libération de l'angle colique gauche, avec rétablissement de la continuité, par laparotomie
HHFA010	Colectomie gauche sans libération de l'angle colique gauche, avec rétablissement de la continuité, par coelioscopie ou par laparotomie avec préparation par coelioscopie
HHFA024	Colectomie gauche avec libération de l'angle colique gauche, sans rétablissement de la continuité, par laparotomie
HHFA006	Colectomie gauche avec libération de l'angle colique gauche, avec rétablissement de la continuité, par laparotomie
HHFA002	Colectomie gauche avec libération de l'angle colique gauche, avec rétablissement de la continuité, par coelioscopie ou par laparotomie avec préparation par coelioscopie
HHFA021	Colectomie totale avec conservation du rectum, sans rétablissement de la continuité, par laparotomie
HHFA005	Colectomie totale avec conservation du rectum, sans rétablissement de la continuité, par coelioscopie ou par laparotomie avec préparation par coelioscopie
HHFA022	Colectomie totale avec conservation du rectum, avec anastomose iléorectale, par laparotomie
HHFA004	Colectomie totale avec conservation du rectum, avec anastomose iléorectale, par coelioscopie ou par laparotomie avec préparation par coelioscopie
HHFA030	Coloproctectomie totale sans rétablissement de la continuité, par laparotomie
HHFA029	Coloproctectomie totale sans rétablissement de la continuité, par coelioscopie ou par laparotomie avec préparation par coelioscopie
HHFA031	Coloproctectomie totale avec anastomose iléoanale, par laparotomie
HHFA028	Coloproctectomie totale avec anastomose iléoanale, par coelioscopie ou par laparotomie avec préparation par coelioscopie
HJFC031	Résection rectosigmoïdienne dépassant le cul‐de‐sac de Douglas, sans rétablissement de la continuité, par coelioscopie
HJFA011	Résection rectosigmoïdienne dépassant le cul‐de‐sac de Douglas, sans rétablissement de la continuité, par laparotomie
HJFA002	Résection rectosigmoïdienne avec anastomose colorectale infrapéritonéale, par laparotomie
HJFA004	Résection rectosigmoïdienne avec anastomose colorectale infrapéritonéale, par coelioscopie ou par laparotomie avec préparation par coelioscopie
HJFA006	Résection rectosigmoïdienne par laparotomie, avec anastomose coloanale par voie anale ou par abord transsphinctérien
HJFA017	Résection rectosigmoïdienne par coelioscopie ou par laparotomie avec préparation par coelioscopie, avec anastomose coloanale par voie anale
HJFA001	Résection rectocolique avec abaissement colique rétrorectal par laparotomie, avec anastomose colorectale par voie anale
HJFA005	Amputation du rectum, par abord périnéal
HJFA007	Amputation du rectum, par laparotomie et par abord périnéal
HJFA019	Amputation du rectum, par coelioscopie ou par laparotomie avec préparation par coelioscopie et par abord périnéal
HJFA014	Exérèse de moignon rectal résiduel, par abord périnéal
HJFC023	Proctectomie secondaire par coelioscopie avec anastomose iléoanale par voie transanale, après colectomie totale initiale
HJFA012	Proctectomie secondaire par laparotomie avec anastomose iléoanale par voie transanale, après colectomie totale initiale
HLFA014	Séquestrectomie hépatique, par laparotomie
HLFA012	Kystectomie ou périkystectomie hépatique, par laparotomie
HLFA002	Résection du dôme saillant de kyste hydatique du foie, par laparotomie
HLFC003	Résection atypique du foie, par coelioscopie
HLFA019	Résection atypique du foie, par laparotomie
HLFC004	Unisegmentectomie hépatique, par coelioscopie
HLFA020	Unisegmentectomie hépatique, par laparotomie
HLFA003	Résection du lobe caudé [de Spigel] [segment I] du foie, par laparotomie
HLFC027	Bisegmentectomie hépatique, par coelioscopie
HLFA009	Bisegmentectomie hépatique, par laparotomie
HLFC032	Trisegmentectomie hépatique, par coelioscopie
HLFA006	Trisegmentectomie hépatique, par laparotomie
HLFC002	Lobectomie hépatique gauche, par coelioscopie
HLFA011	Lobectomie hépatique gauche, par laparotomie
HLFC037	Hépatectomie gauche, par coelioscopie
HLFA018	Hépatectomie gauche, par laparotomie
HLFA007	Hépatectomie gauche élargie au lobe caudé [de Spigel] [segment I], par laparotomie
HLFA017	Hépatectomie droite, par laparotomie
HLFA004	Hépatectomie droite élargie au lobe caudé [de Spigel] [segment I], par laparotomie
HLFA005	Lobectomie hépatique droite [Hépatectomie droite élargie au segment IV], par laparotomie
HLFA010	Hépatectomie centrale, par laparotomie
HMFA009	Résection de la voie biliaire principale pédiculaire avec anastomose biliodigestive, par laparotomie
HMFA010	Résection de la voie biliaire principale pédiculaire et intrapancréatique avec anastomose biliodigestive, par laparotomie
HMFC004	Cholécystectomie, par coelioscopie
HMFA007	Cholécystectomie, par laparotomie
HMFA006	Cholécystectomie par coelioscopie, avec cholédochoduodénostomie par laparotomie
HMFA002	Cholécystectomie avec cholédochogastrostomie ou cholédochoduodénostomie, par laparotomie
HMFA005	Cholécystectomie par coelioscopie, avec cholédochojéjunostomie par laparotomie
HMFA001	Cholécystectomie avec cholédochojéjunostomie, par laparotomie
FFFA002	Splénectomie partielle, par laparotomie
FFFC001	Splénectomie totale, par coelioscopie
FFFA001	Splénectomie totale, par laparotomie

### A.2 Stays with confirmed related infectious complication

Associated diagnosis ICD‐10 code: T814*

### A.3 Stays with probably related infectious complication

Associated diagnosis ICD‐10 code: T818*, T819* ou T827*

+ infectious complication possibly linked to surgical procedure

Associated diagnosis ICD‐10 code : A04*, A09*, A40*, A41*, A49*, J12* à J18*, J20*, J22*, J690*, J85, J86*, J952*, J960*, K560*, K561*, K564*, K566*, K567*, K63* without K635, K65*, K750*, K832*, K833, K91*, L02*, L03*, L04*, L08*, N300*, N303*, N308*, N309*, N34*, N730*, N732*, N733*, N735*, N737*, N738*, N739*, R572*, R650*, R651*

### A.4 Stays with other non infectious complication (also called other complication)

Associated diagnosis ICD‐10 code: T810*, T813*, R571*, R652*, R653*
